# Nonsurgical management of endodontic mishaps in a case of radix entomolaris

**DOI:** 10.4103/0972-0707.58345

**Published:** 2009

**Authors:** Pragati Mirikar, Arvind Shenoy, Goud K Mallikarjun

**Affiliations:** Department of Conservative Dentistry and Endodontics, Bapuji Dental College and Hospital, Davangere - 577 004, Karnataka, India

**Keywords:** Furcal perforation, mineral trioxide aggregate, radix entomolaris, separated instrument, ultrasonics

## Abstract

Anatomic variations can significantly contribute to the incidence of endodontic mishaps. Perforations and separated instruments form the bulk of such mishaps. Furcal perforations are undesired complications of endodontic treatment, which result in the loss of integrity of the root and further destruction of the adjacent periodontal tissues. Mineral trioxide aggregate (MTA) is a promising material that has been successfully used to repair perforations. This clinical case demonstrates the use of MTA as a repair material for furcal perforation due to an iatrogenic error in radix entomolaris in the mandibular first molar. It also describes the application of ultrasonic technique in the retrieval of separated instrument from the same. Both clinical and radiographic follow-up showed a stable condition without any probing defect, ongoing root resorption, or furcal pathosis.

## INTRODUCTION

Endodontics is the preparatory discipline in which the treatment focuses on conservative or prosthetic restoration of a tooth. Maintaining the integrity of natural dentition is essential for fully functional and esthetic conditions.[1] One of the major undesired complications of endodontic and restorative treatment procedures is the accidental perforation of the roots or pulp chamber floor.[2,3] Root perforations are artificial openings in root walls created by boring, piercing, cutting, or resorption that result in a communication between the pulp space and periodontal tissues.[4] The result is a chronic inflammatory reaction of the periodontium (characterized by the formation of granulation tissue) that can lead to irreversible loss of attachment or loss of the tooth.[5] Such perforations are managed surgically or nonsurgically depending on the particular characteristics of the case.[6]

An ideal endodontic repair material should exhibit certain characteristics as follows: It should maintain a hermetic seal; it should be insoluble in tissue fluids, dimensionally stable, nonresorbable and also must exhibit biocompatibility, if not bioactivity.[7,8] Mineral trioxide aggregate (MTA) is a biomaterial that has been investigated for endodontic applications since the early 1990s. MTA was first described in the dental literature in 1993 and was given approval for endodontic use by the U.S. Food and Drug Administration in 1998.[7] The unique physical characteristics of MTA allow for superior marginal adaptation. Further, its sealing ability in hermetic environment along with the osteo/cemento conductive attributes makes it an excellent material for perforation repair.[8,9] MTA may be the ideal material for using against bones because it is the only material that consistently allows for the overgrowth of cementum and formation of bone. Further, it may also facilitate the regeneration of the periodontal ligament.[10] This concept has also been proved by Koh *et al*. by using a human osteoblast model. He found that MTA stimulated the upregulation of cytokines, such as interleukin-1, interleukin-1β, and interleukin-6, which are involved in bone turnover.[11] In addition, the inherent hydrophilic properties of MTA allow the repair material to set in a wet environment and adequately seal the perforation site.[12]

It is known that the mandibular first molar can display several anatomical variations. An additional third root, first mentioned in the literature by Carabelli is called radix entomolaris (RE). This supernumerary root is located distolingually in mandibular molars, mainly the first molars.[13] The prevalence of RE has been reported to be as low as 0.2% in Indian population, whereas it is higher than 5% (even up to 40%) in population with Mongolian traits.[14] Several refinements during radiographic interpretation, access cavity preparation and cleaning and shaping are required in order to avoid procedural errors during the successful endodontic management of RE.[13]

Every clinician who has practiced endodontics has experienced the dilemma of broken or separated instruments.[15] Various factors contributing to breakage have been identified and the prognosis of leaving versus removing broken instruments from the canal have been discussed in the literature.[15,16] Today, separated instruments can be removed because of technological advancements in vision, ultrasonic instrumentation, and microtube delivery methods.[15–18]

This article presents a clinical case report dealing with the utilization of MTA in successfully repairing furcal perforation along with the use of ultrasonic tips to retrieve separated instrument in a case of RE in mandibular first molar.

## CASE REPORT

**Figure 1:**
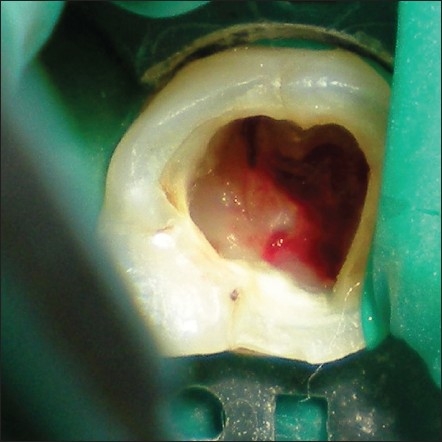
Mandibular first molar, with iatrogenic furcation perforation on the lingual aspect

**Figure 2:**
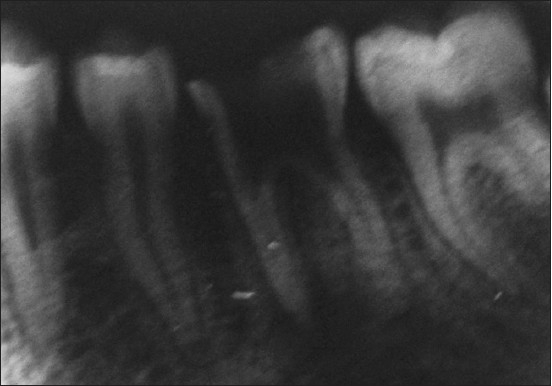
Radiographic evidence of perforation defect extending into the furcation area

A 16-year-old male who presented with accidental furcal perforation, which had occurred during the access preparation for root canal treatment of tooth no. 36, was referred immediately to the Department of Conservative Dentistry and Endodontics from a private dental clinic. On examination of the endodontic cavity, an irregular perforation defect was evident on the lingual aspect at the level of pulpal floor, which was accompanied by bleeding [Figure 1]. The decision to nonsurgically manage the perforation using MTA was taken with the patient's consent. Furcal perforation was confirmed by periapical radiograph of tooth no. 36, which revealed osseous breakdown at the furcation and the presence of a supernumerary root was also suggested [Figure 2].

**Figure 3:**
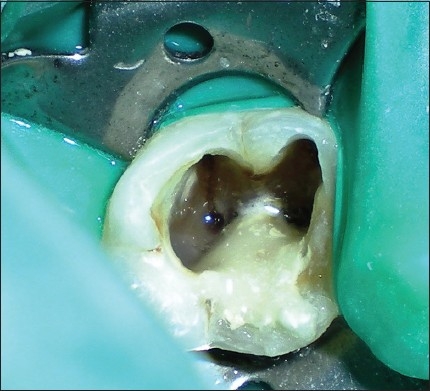
Repair of the perforation defect using mineral trioxide aggregate barrier material

On clinical examination, there was no evidence of attachment loss. Access cavity was refined to locate the canal orifices along with the orifice of distolingually located RE. The canals were negotiated and hemostasis was achieved using calcium hydroxide.[19] The defect was disinfected using 2.5% NaOCl,[18,20] and the operative site was prepared to receive the restorative material. Cotton pellets moistened in saline were placed in the root canals, and the perforation was repaired using MTA (ProRoot® MTA System; DENTSPLY, Tulsa) mixed with sterile saline[18] as suggested by the manufacturer. MTA was carried with an amalgam carrier to the repair site and was condensed against the defect using a MTA plugger through the coronal access cavity in small increments. MTA was gently packed against the dentinal wall by using a damped cotton pellet [Figure 3]. Barrier material was covered with a cotton pellet moistened with distilled water, and Cavit temporary restoration material (3M ESPE, St. Paul, Minn.) was placed to seal the endodontic cavity.[18]

**Figure 4:**
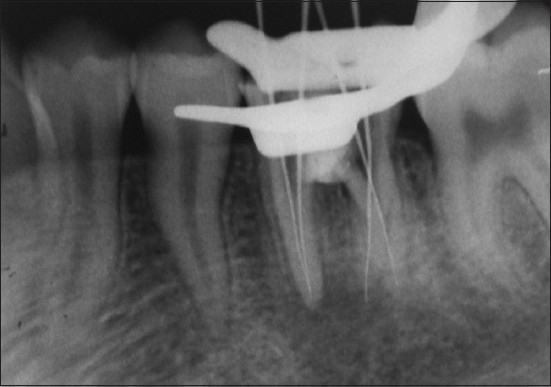
Working length determination radiograph

At the next appointment, the setting of MTA was checked with a DG-16 probe, and the root canals were explored with ISO no. 10, 15 and 20 K-files (DENTSPLY Maillefer, Ballaigues, Switzerland). After determining the tentative working length and achieving a straight line access, the shaping of the initial two-thirds of all the canals was performed with ProTaper instrument system (DENTSPLY/Maillefer, Ballaigues, Switzerland) using crown- down technique described by Marshall and Papin.[21] The working length was electronically determined (Elements Diagnostic Unit, SybronEndo) and also confirmed radiographically. The presence of RE was evident on the working length radiograph [Figure 4]. EDTA paste (Glyde, DENTSPLY Maillefer, Ballaigues Switzerland) was used for lubrication. The canals were debrided and disinfected using copious amounts of 2.5% NaOCl (22). Middle and apical thirds were prepared, and this was followed by apical gauging using hand instruments – nickel-titanium NiTiFlex files[22] – during which the separation of size 30 file occurred in the extra root (RE).

**Figure 5:**
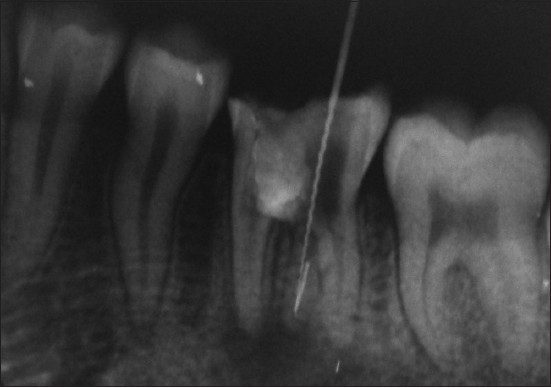
Radiograph showing inability to bypass the separated instrument

The presence of the separated instrument was confirmed using a radiograph, which resulted in reducing the working length to 1.5-2 mm [Figure 5].

**Figure 6:**
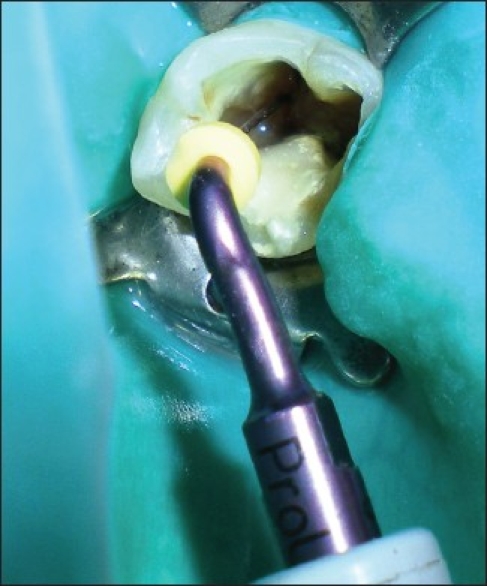
Use of ProUltra Endo tip to remove the separated instrument

The “bud” of Gates Glidden Drills no. 2 (DENTSPLY Maillefer, Ballaigues Switzerland) was “modified” and used to create a circumferential staging platform. After unsuccessful attempts to bypass the file, trephining was done by with using K-files ISO size 6, 8, 10, 15 and 20.[15,18] Titanium Ultrasonic instrument, ProUltra Endo 7 (ProUltra ENDO Instruments, DENTSPLY International), in synergism with piezoelectric generator (P5, DENTSPLY International) was used to allow intimate contact with the instrument fragment with an unobstructed line of sight into the canal [Figure 6]. A stream of compressed air and water at low power setting was used, which lead to the unwinding and spinning of the instrument out of the canal.[15,16]

**Figure 7:**
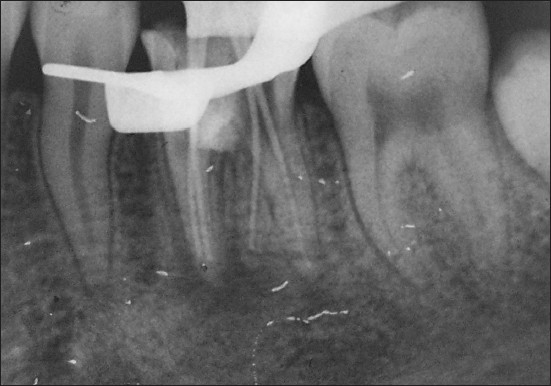
Master cone selection radiograph evidencing successful removal of separated instrument

**Figure 8:**
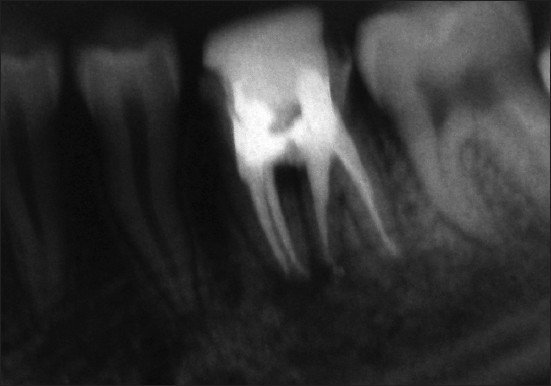
Post obturation radiograph

After thorough cleaning and shaping, selection of Gutta-percha master cone was confirmed by using a radiograph [Figure 7]. Obturation was performed using cold lateral compaction technique using AH Plus sealer (De Trey DENTSPLY, Konstanz, Germany) [Figure 8].[23] Permanent coronal seal and core build up was done using composite resin. On a follow up examination after 15 days, 1 month and 2 months, satisfactory healing was evident both clinically and radiographically.

## DISCUSSION

The presence of a separate RE in the first mandibular molar is associated with certain ethnic groups. In African populations, a maximum frequency of 3% is found,[24] while in Eurasian and Indian populations the frequency is less than 5%.[14] The etiology behind the formation of the RE is unknown and can be related to external factors during odontogenesis or to the penetrance of an atavistic gene or polygenetic system. It is hypothesized that the presence of RE adds to the stability of molars by providing an increased surface area of attachment to the alveolus.[25] This anatomic structure has important clinical implications as their anatomical knowledge aids in avoiding endodontic mishaps and procedural errors.[13]

The four defining characteristics of a perforation that always occur in combination are level, location, size and time. Regardless of the etiology, the perforation should be repaired as soon as possible to discourage further loss of attachment and to prevent periodontal pocket formation.[26] The perforation was repaired before cleaning and shaping so as to control bleeding into the canal, confine irrigation, and facilitate obturation.[18]

Selecting an appropriate restorative material is very necessary to successfully repair a perforation. Different materials have been used for the nonsurgical repair of perforations with varying degrees of success.[1] MTA has many clinical applications and represents an extraordinary breakthrough for managing radicular repairs.[8–10] It exhibits superior biocompatibility and can be used as a nonabsorbable barrier and restorative material. Furthermore, MTA can be used as a sole barrier (without bio-inert matrices) against which other materials can be condensed.[6]

MTA is a fine powder primarily composed of tricalcium silicate, tricalcium aluminate, tricalcium oxide, and silicon oxide that forms a colloidal gel on hydration, which solidifies in approximately 3 hours.[12] Therefore, when used as a root repair material, moisture must be provided from the internal aspect of the root (using a moist cotton pellet). The choice of MTA was made as there was no communication with the gingival sulcus. Reports have strongly suggested that the favorable biologic performance exhibited by MTA materials is due to the formation of hydroxyapatite when these materials are exposed to physiologic solutions.[27,28] Studies have reported its cement-inductive effect and have shown that MTA is the material often observed with bone apposition.[10,28,29] When used as a root end filling material in dogs it was associated with less inflammation, cementum formation over MTA, and regeneration of the periradicular tissues almost to the normal pre-experimental level.[10,29]

The factors influencing broken instrument removal should be identified and taken into consideration while attempting such cases.[30] The ability of nonsurgical access to remove a broken instrument will be influenced by the diameter, length and position of obstruction within the canal.[15,18,31] Additionally, the potential for safely removing a broken instrument is guided by anatomy, including the diameter, length and curvature of the canal. Importantly, the potential for safely removing a broken instrument is limited by root morphology, including the circumferential dimensions and thickness of dentin and depth of external concavity.[15,31]

Before commencing with the efforts to remove a broken instrument, the clinician should thoughtfully observe different horizontally angulated radiographs.[16] Coronal access is the first step in the removal of broken instruments. Special attention should be directed towards flaring the axial wall that approximates the canal holding the broken instrument in efforts to subsequently improve the micro-sonic techniques below the orifice. A number of techniques may be employed in flaring the canal coronal to an intracanal obstruction. However, the predictable and safe way is to initially use hand files, small to large, followed by sequential use of Gates Glidden (GG) drills (DENTSPLY Maillefer, Ballaigues Switzerland). GG sizes 1 to 4 are most commonly used in multi-rooted teeth to remove fractured instruments. They should be cautiously used in approximation to the obstruction and care should be ensured to use them away from the furcation.[15,16]

Radicular access is the second step required in the successful removal of broken instrument. At times, when an ultrasonic instrument is introduced into a pre-enlarged canal, its activated tip does not have sufficient space lateral to the broken file segment to initiate trephining procedures. Ideally, radicular access should be performed in a way that the canal is pre-enlarged and shaped to the same diameter as that if there was no broken instrument obstructing the canal.[15,16]

To facilitate excellent visibility of an intra-radicular obstruction, the canal should be vigorously flushed and thoroughly dried before beginning ultrasonic procedures. An ultrasonic generator should provide a broad range of power, precise adjustment within the lower settings and electrical feedback to regulate amplitude and safe tip movement.[15,18] All ultrasonic work below the canal orifice is to be done in suppressed stream of water or completely dry[15] so that the clinician has a constant visualization of the energized tip against the broken instrument. Micro-sonic techniques, as advocated for removal of separated instruments, do not generally generate heat to an extent that it harms the attachment apparatus.[15] In longer and thinner roots, where the space is restricted, abrasively coated zirconium nitride or an appropriately sized titanium instrument is chosen. Titanium instruments provides safety while triphining deep into the canal. Typically during ultrasonic use, gently wedging the energized tip between the tapered file and the canal wall several times causes the broken instrument to abruptly “jump out” of the canal.[15,16]

In cases presenting with anatomical variations, such as RE, an integrated endodontic protocol should be followed. This should be done by giving thorough consideration to locate the distolingually located orifice during endodontic access cavity preparation as well as to distolingual inclination and radicular curvature of the supernumerary root. As a result, the separation of the instrument during radicular preparation can be prevented.[13] The best way to avoid fractured instrument is prevention. Adhering to proven concepts and utilizing safe techniques during root canal preparation procedures will eliminate most of the instrument fractures.[15,18]

## CONCLUSION

Refinements in endodontic cavity preparation and radicular inclinations should be given due considerations in teeth presenting anatomical variations such as radix entomolaris so as to prevent procedural accidents during various endodontic and restorative procedures. Perhaps, the most important factors central to successful instrument removal are knowledge, training and competency in selecting the best developed and proven technologies and techniques available at present. However, the best antidote for a broken file is prevention.
